# Comparative fitness analysis of D-cycloserine resistant mutants reveals both fitness-neutral and high-fitness cost genotypes

**DOI:** 10.1038/s41467-019-12074-z

**Published:** 2019-09-13

**Authors:** Dimitrios Evangelopoulos, Gareth A. Prosser, Angela Rodgers, Belinda M. Dagg, Bhagwati Khatri, Mei Mei Ho, Maximiliano G. Gutierrez, Teresa Cortes, Luiz Pedro S. de Carvalho

**Affiliations:** 1 Mycobacterial Metabolism and Antibiotic Research Laboratory, The Francis Crick Institute, 1 Midland Road, London, NW1 1AT UK; 2 Bacteriology Division, National Institute for Biological Standards and Control (MHRA-NIBSC), Blanche Lane, South Mimms, Potters Bar, Herts, EN6 3QG UK; 3 Host-Pathogen Interactions in Tuberculosis Laboratory, The Francis Crick Institute, 1 Midland Road, London, NW1 1AT UK; 4 Department of Infection Biology, Faculty of Infectious and Tropical Diseases, London School of Hygiene and Tropical Medicine, London, WC1E 7HT UK; 5 Present Address: Cancer Research UK Manchester Institute, The University of Manchester, Alderley Park, Manchester, SK10 4TG UK

**Keywords:** Antibiotics, Antimicrobial resistance, Bacterial genomics, Pathogens

## Abstract

Drug resistant infections represent one of the most challenging medical problems of our time. D-cycloserine is an antibiotic used for six decades without significant appearance and dissemination of antibiotic resistant strains, making it an ideal model compound to understand what drives resistance evasion. We therefore investigated why *Mycobacterium tuberculosis* fails to become resistant to D-cycloserine. To address this question, we employed a combination of bacterial genetics, genomics, biochemistry and fitness analysis in vitro, in macrophages and in mice. Altogether, our results suggest that the ultra-low rate of emergence of D-cycloserine resistance mutations is the dominant biological factor delaying the appearance of clinical resistance to this antibiotic. Furthermore, we also identified potential compensatory mechanisms able to minimize the severe fitness costs of primary D-cycloserine resistance conferring mutations.

## Abstract (web summary)

D-cycloserine (DCS) has been used for decades to treat *Mycobacterium tuberculosis* (Mtb) but resistance is rarely observed in clinical isolates. Here, the authors report ultra-low rate of emergence of resistance mutations as the underlying mechanism of DCS resistance evasion in Mtb.

## Introduction

The number of human infections caused by bacteria resistant to antibiotics has dramatically increased in the last decades^1–3^. Currently, almost all drug classes in clinical use are matched by evolved mechanisms of resistance in bacteria^4,5^. Some of these bacteria are pan-resistant and cannot be treated with any approved drugs^6^. While we understand very well how these antibiotics work and how antibiotic resistance works in many cases, we still fail to recognize how some drugs select for resistance in months while others need several decades, which is defined here as resistance evasion^7,8^.

As for all other human pathogens, *Mycobacterium tuberculosis*, the etiological agent of human tuberculosis (TB), has acquired mutations in its genome and evolved resistance mechanisms to almost all drugs that it has encountered during chemotherapy^9^. Currently, the genetic basis for first-line and second-line antibiotic resistance in *M. tuberculosis* is well characterized, with a few exceptions, one of them being the antibiotic D-cycloserine (DCS)^10^. DCS belongs to the core second-line treatment group B listed in the WHO guidelines for treatment of multidrug and extensively drug-resistant-TB (MDR/XDR-TB)^11^. The mechanism of DCS action has been well studied since its discovery in the 1950s. In all bacteria tested, DCS inhibits two enzymes of the D-Ala-D-Ala branch of peptidoglycan biosynthesis: alanine racemase (Alr) and D-Ala:D-Ala ligase (Ddl)^12,13^. In *M. tuberculosis*, it has been established that despite DCS still inhibiting both targets, the principal mechanism of bacterial death is inhibition of Ddl (encoded by the *ddlA* gene), instead of inhibition of Alr (encoded by the *alr* gene)^14,15^. This conclusion is supported by earlier independent work which demonstrates a high catalytic excess of alanine racemase in *Mycobacterium smegmatis*^16^.

Surprisingly, until very recently there were no well-defined, associated mutations conferring DCS resistance in the clinic. As DCS inhibits two essential targets, it is expected that it will display a low frequency of high-level resistant isolates emerging in the clinic^17^. In addition, there is no well-defined critical concentration (CC) for DCS making the assignment of resistant isolates problematic and often depending on different epidemiological cutoffs^18^. Still, it has been described that in the fast-grower, free-living *M. smegmatis*, artificial overexpression of both Alr and Ddl result in DCS resistance^19,20^. Furthermore, a point mutation in the *cycA* gene, encoding the DCS transporter, is responsible for the natural DCS resistance of *Mycobacterium bovis* BCG^21^. Recently, whole-genome sequencing (WGS) data from clinical isolates including MDR and XDR-TB strains identified non-synonymous single-nucleotide polymorphisms (SNP) in the *alr* gene with the possibility of some of them conferring low-level DCS resistance^22–24^. However, whether these SNPs are relevant DCS-resistance mutations in *M. tuberculosis* remains unclear. Given its activity and lack of reported resistance in strains infecting humans, DCS has been called “the cornerstone option” for treating drug-resistant TB cases^25^. In August 2018, WHO released a recent report to highlight changes made for the treatment of MDR-TB. It is now recommended by WHO that DCS along with clofazimine will be added to all regimens for treating MDR-TB patients^11^. Even though DCS is only been used to treat multidrug-resistant TB infections (which account for almost half a million cases per year), this feature makes DCS the only antibiotic that has been used in humans for almost seven decades that has evaded resistance selection in bacterial populations^26,27^. From a practical standpoint, the molecular and cellular determinants for DCS resistance–evasion are highly attractive features that if understood could be incorporated into antibiotic drug discovery programs aimed at developing superior antimicrobial agents for the treatment of TB and other infectious diseases.

In this study, we confirm the ultra-low rate of emergence of spontaneous mutations conferring DCS resistance in *M. tuberculosis*. These mutants display significant fitness cost during infection, but not in vitro. We also identified compensatory mechanisms that are in place to cope with the initial fitness cost of the DCS-resistant (DCS^R^) mutants. Finally, we also characterized the molecular and cellular basis of DCS resistance, with Alr overexpression or Alr mutation being sufficient to resist to DCS treatment. Altogether, these results suggest that the lack of clinical resistance to DCS is likely to be due to the ultra-low rate of emergence of DCS-resistance mutations, as no-fitness phenotypes can appear and be propagated during infection.

## Results

### Low abundance of SNPs in DCS-related genes

To identify possible clues of DCS resistance in *M. tuberculosis*, we first analyzed the distribution and frequency of SNPs in the DCS target genes *alr, ddlA, cycA* as well as the *ald* gene, which has been recently suggested to cause low-level DCS resistance^24^. We screened a published data set comprising 1601 genomes of *M. tuberculosis* clinical isolates^28^ and compared it to the polymorphisms found in *rpoB* and *gyrA*, associated with drug resistance to rifampicin^29^ and fluoroquinolones^30^, respectively. We identified scarce non-synonymous SNPs in the *ddlA* (0.80%) and *alr* genes (0.98%), and a slightly higher frequency of non-synonymous SNPs in the *cycA* (1.26%) and *ald* genes (1.52 %) when compared with the *gyrA* and *rpoB* (1.99 and 1.85%, respectively) (Supplementary Fig. 1 and Supplementary Data 1). The presence of *rpoB* mutations could be associated with rifampicin mono-resistance or could be indicative of pre-MDR strains. It is also worth mentioning that in this setting pre-existing fluoroquinolone mono-resistance could also be present^31^. Therefore, these results demonstrate a low degree of SNPs in the genes encoding DCS targets, in agreement with lack of DCS resistance in clinical isolates. Together these data demonstrate that while we have limited information on the potential genes involved in DCS resistance, there is no experimentally validated phenotypic, cellular, and molecular data supporting their role in DCS resistance, and thus we decided to generate and study lab-derived spontaneous DCS-resistant mutants.

### DCS has an extremely low mutation rate

To investigate the cause of such low-resistance incidence in *M. tuberculosis*, we first examined the rate of spontaneous mutations conferring resistance to DCS, by carrying out fluctuation analysis experiments, initially employed by S. Luria and M. Delbrück^32^. Fluctuation analysis is a parallel evolution experiment that allows the distinction between spontaneous versus induced mutations and allows the determination of the mutation rates. Using this approach, we calculated an ultra-low rate of emergence of spontaneous mutations conferring DCS resistance in *M. tuberculosis* (in the order of 10^−11^ mutations per bacterium per cell division, Table 1), and that DCS does not induce mutagenesis. This result is in agreement with the only other report of the mutation rates of DCS with *M. tuberculosis*^33^. This ultra-low rate of emergence of DCS-resistance mutations is consistent with DCS engaging two lethal targets in *M. tuberculosis*, Alr and Ddl, and represents an important barrier for the selection of resistance. Another valid interpretation of this ultra-low rate of DCS resistance is independent of the number of targets engaged per se, and encompasses a finite number of polymorphisms that can confer DCS resistance. In contrast, the mutation rates for isoniazid and rifampicin, the two most commonly used anti-tubercular drugs were calculated at 4.8 × 10^−8^ and 3.2 × 10^−9^ mutations per bacterium per cell division, respectively (Table 1). Nevertheless, under antibiotic pressure the mutation rate for DCS might be different, as suggested from a recent study reporting DCS clinical resistance^22,24^.

**Table 1:** Mutation rates of DCS, RIF, and INH as calculated from fluctuation assays

Strain	Drug	Concentration (μg/mL)	Mutation rate (μ) per bacterium per cell division
H37Rv	DCS	100	1.63–1.79 × 10^−11^
H37Rv	DCS	64	1.25–3.22 × 10^−10^
H37Rv	RIF	2	3.21 × 10^−9^
H37Rv	INH	1	4.86 × 10^−8^

### Alr is the sole contributor to DCS resistance in *M. tuberculosis*

**Fig. 1:**
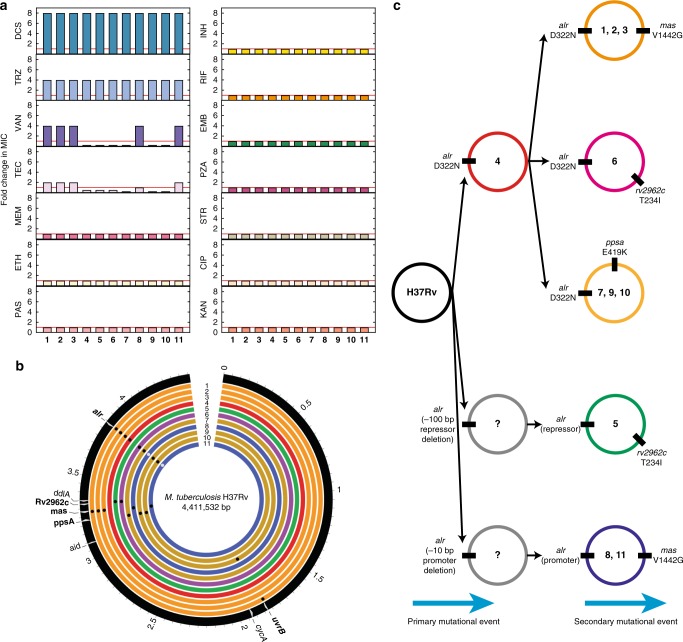
Drug susceptibility profile of the DCS^R^ strains, distribution of DCS^R^ mutations and evolution of resistant strains. **a** Drug susceptibility pattern of DCS-resistant mutants (MIC_90_ fold change DCS^R^/parent strain). Drug abbreviations used: DCS D-cycloserine, TRZ terizidone, VAN vancomycin, TEC teicoplanin, MEM meropenem, INH isoniazid, RIF rifampicin, EMB ethambutol, PZA pyrazinamide, STR streptomycin, CIP ciprofloxacin, KAN kanamycin, ETH ethionamide, PAS para-aminosalicylic acid. The MIC_90_ for DCS of the parent, DCS^S^ strain was 6.25 µg mL^−1^. The data are representative of three independent experiments. **b** Genome visualization illustrating the SNPs identified in the DCS^R^ strains compared with the parent, DCS^S^ strain. Moving from the outer to innermost ring, genome reference is indicated in black, with genes harboring SNPs or representing DCS targets highlighted in white, followed by DCS^R^ strains 1–11. Presence of non-synonymous SNPs is represented with black dots, while SNPs within the promoter region of the *alr* gene are colored in gray. Names of genes with identified SNPs have been highlighted in bold. Mutants have been color-coded, based on the types and polymorphisms observed in the genome. Circular map was generated using Circos^53^. **c** Schematic representation of evolution of DCS resistance. Circles represent the genomic DNA of each strain and numbers refer to the specific mutant ID. Source data are provided as a Source Data file

To investigate the likelihood of secondary compensatory mutations and experimentally define the mechanisms responsible for DCS resistance in *M. tuberculosis*, we isolated and characterized 11 spontaneous DCS^R^, obtained independently. All eleven mutants are eightfold more resistant to DCS when compared with the parent strain (Fig. 1a). In line with these results, all 11 mutants were also fourfold more resistant to terizidone (TRZ), a better tolerated DCS pro-drug that contains two equivalents of DCS per molecule^34^. In order to identify the genomic mutations conferring DCS resistance, we whole-genome sequenced all 11 DCS^R^ strains along with the parental *M. tuberculosis* H37Rv strain (Supplementary Data 2). A total of 85 nonredundant SNPs were identified across the resistant isolates, when compared against the reference genome of *M. tuberculosis* H37Rv. After excluding SNPs within repetitive regions, a total of 11 SNPs were targeted for further analysis (Fig. 1b; Supplementary Data 3). Among the genes known to be potential DCS targets or associated with DCS resistance, we only detected mutations in the *alr* gene (Fig. 1b, c). Eight out of the 11 mutants shared a non-synonymous SNPs in position 3840391 (C > T), creating an aspartate (D) to asparagine (N) substitution at codon 322 of the Alr protein. Furthermore, DCS^R^8 and DCS^R^11 shared a mutation located within the promoter region (C 3841405A) of the *alr* gene. This mutation changes the −10 promoter region from a non-TANNNT −10 motif to an extended −10 promoter consensus sequence^35^, suggesting possible transcriptional changes. In addition, we detected a 142 bp deletion upstream of the *alr* promoter in DCS^R^5, possibly in a region where a transcriptional regulator might bind. In silico analysis of transcriptional factor-binding site indicates that no known transcriptional regulators bind to this region, suggesting it could be a novel regulator. After screening of these two SNPs in the Coll et al.^28^ dataset, only a single occurrence of the first SNP (C 3840391T; D322N) was reported in a Russian strain belonging to lineage 1. Based on the pattern of non-synonymous SNPs detected among the mutant strains, we arranged them into six different groups (Fig. 1b, c). Furthermore, mutations in the promoter or putative repressor site of *alr* seem to have evolved in parallel, as all the secondary mutations found in these strains are also present in the *alr* D322N mutant strains. These data highlight the central role of *alr* in DCS resistance in *M. tuberculosis* in vitro, which is in strict agreement with the few identified and phenotypic validated clinical DCS-resistant strains.

### DCS^R^ mutants do not exhibit cross-resistance

We next tested the extent of resistance and whether these mutants display any cross-resistance against other antitubercular drugs. Minimal inhibitory concentrations (MIC_90_) were evaluated for all first-line and second-line antitubercular drugs, as well as other peptidoglycan targeting drugs. Interestingly, these mutants showed differential susceptibility patterns when tested against vancomycin (VAN) and teicoplanin (TEC). VAN and TEC are glycopeptide antibiotics that bind to the terminal D-Ala:D-Ala moiety of the pentapeptide stem of peptidoglycan and prevent the cross-link of the peptidoglycan chains. Mutants DCS^R^1, DCS^R^2, DCS^R^3, DCS^R^8, and DCS^R^11 were fourfold more resistant to VAN, whereas the rest of the DCS^R^ mutants were fourfold more susceptible when comparing with parent strain (Fig. 1a). It seems that the DCS^R^ strains containing the *mas* gene (V1442G) SNP are more resistant to VAN and TEC than the other strains. The *mas* gene encodes a polyketide synthase involved in mycocerosic acid synthesis, a part of the highly lipophilic outer membrane lipid phthiocerol dimycocerosate (PDIM). In line with our results, changes in PDIMs have been previously shown to affect the susceptibility of *M. tuberculosis* strains to VAN^36^. Importantly, not all DCS^R^ mutants have altered PDIMs (Supplementary Fig. 2). The sensitivity to glycopeptides, triggered by resistance to DCS also highlights the collateral implications that certain antibiotic resistant strains may have with other antibiotics^37^. Of broad interest, the fact that a single SNP can confer VAN resistance in *M. tuberculosis* is highly unusual. For example, in glycopeptide-resistant enterococci, mobile elements containing at least four but up to seven genes are required to cause VAN resistance^38,39^. Furthermore, we also performed MIC assays using the automated BD BACTEC™ MGIT instrument as it is currently used for drug susceptibility testing and determination of resistance in clinical strains. In this assay the MIC value of the parent H37Rv strain was 8 µg mL^−1^, whereas the MIC value for the DCS^R^ mutants was 64 µg mL^−1^. As there is no acceptable critical concentration of DCS, using the most recently defined epidemiological cutoff of 64 µg mL^−1^ will classify these mutants as DCS resistant^18^. Importantly, no cross-resistance to any first-line and second-line drugs was observed in the 11 DCS^R^ mutants (Fig. 1a), indicating that these strains bear mutations that are specifically linked to DCS resistance.

### DCS^R^ mutants are fit in vitro

**Fig. 2:**
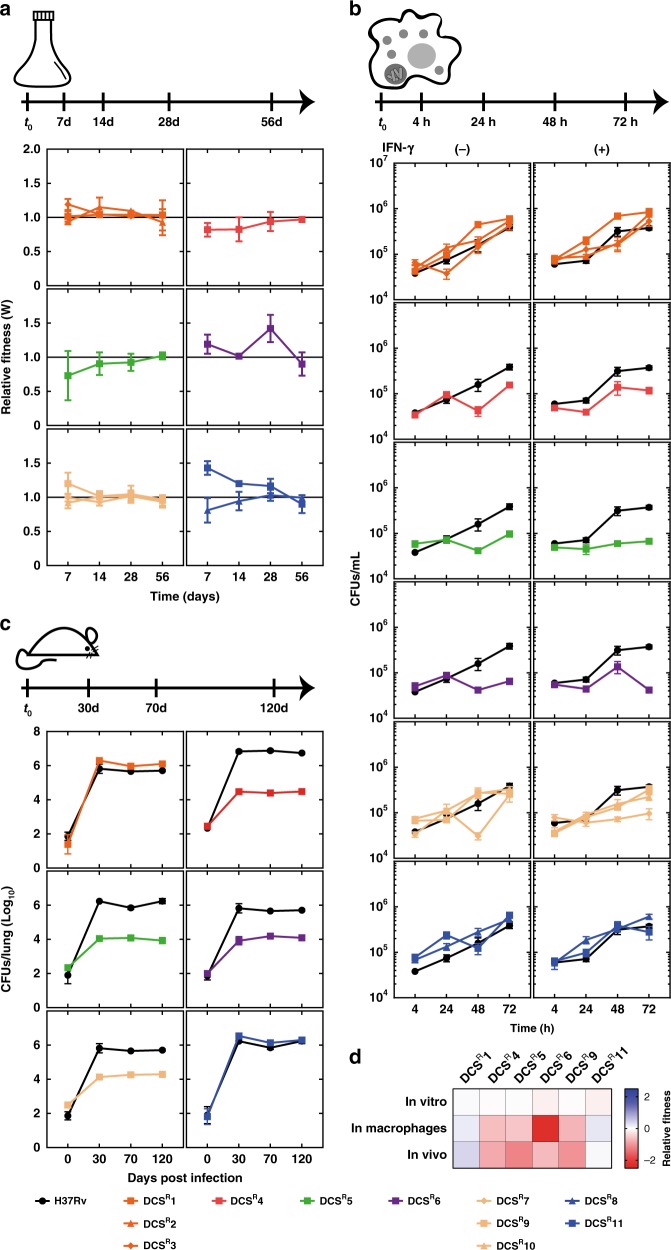
Fitness cost kinetics of DCS^R^ mutants. **a** Relative fitness of DCS^R^ mutants calculated using competition assays in vitro. DCS^R^ mutants are grouped according to their mutations and the relative fitness is followed over time. Parent strain relative fitness W = 1. The data are representative of three independent experiments. The data points are representative of the mean, whereas error bars represent standard error SEM, *n* = 2. **b** Relative fitness of DCS^R^ mutants obtained during macrophage infection. DCS^R^ mutants are grouped according to their mutations. Experiments were carried out with human blood-derived monocytes differentiated into macrophages. Bacterial uptake and survival inside naive or interferon-gamma-activated macrophages is compared over time with the wild-type strain (black dots and line). The data shown are from one experiment representative of three independent experiments, where each macrophage culture originated from at least three blood donors and were pooled prior to infection to minimize donor variability. The data points are representative of the mean whereas error bars represent standard error SEM, *n* = 3. **c** Assessment of fitness cost associated with DCS resistance in the mouse model of infection. DCS^R^ mutants (1, 4, 5, 6, 9, and 11) and parent strain were used in a low-dose aerosol infection murine model (using C57BL/6 mice). The CFUs obtained from mouse lungs are shown for days 0, 30, 70 and 120 post infection. The data for DCS^R^4 are representative of two independent experiments, whereas for DCS^R^ mutants (1, 5, 6, 9, and 11) are from a single infection experiment. Five mice were infected per strain per timepoint. The data points are representative of the mean, whereas error bars represent standard error SEM, *n* = 5. **d** The relative fitness calculated for representative DCS^R^ mutants (1, 4, 5, 6, 9, and 11) at the endpoint in vitro (day 56) competition assays, macrophages infection assays (day 3), and in vivo experiments (day 120), compared with the parent strain. Source data are provided as a Source Data file

We next sought to investigate the potential fitness cost that these mutations might confer, which if high, could explain the scarcity of DCS-resistant *M. tuberculosis* strains in the population. Given the slow-growing nature of *M. tuberculosis*, we carried out these experiments as a function of time, by measuring fitness kinetics, instead of estimating fitness at a single arbitrarily chosen time. We examined fitness in vitro, in standard Middlebrook 7H9 medium, using a direct competition assay; inside interferon-gamma-stimulated or naive human monocyte-derived macrophages, and finally, in vivo in a mouse model of infection (Fig. 2). In vitro, DCS^R^4, the mutant that solely contains the primary mutation D322N in Alr exhibited a very weak, transient fitness cost (at days 7 and 14), which was not statistically significant. This difference was lost by the end of the assay (day 56) (Fig. 2a). Similar fitness kinetics were also observed with DCS^R^5, the mutant having a deletion upstream of the *alr* gene. Furthermore, the occurrence of the *mas* V1442G in DCS^R^1, DCS^R^2, DCS^R^3, as well as the occurrence of *ppsA* E419K in the DCS^R^7, DCS^R^9, and DCS^R^10 do not seem to bear any possible fitness cost in addition to the *alr* D322N SNP (Fig. 2a). Moreover, mutants DCS^R^8 and DCS^R^11 containing the promoter SNP did not exhibit any noticeable fitness cost over time. The fact that there were no DCS-resistant isolates obtained with only mutations in the promoter region of *alr* indicates that the original mutation might lead to a high-fitness cost phenotype, and thus either mutations in *mas* or *rv2962c* had to be established to provide compensatory effects and survival of these strains prior to promoter changes. DCS^R^7, DCS^R^9, and DCS^R^10 have an additional mutation at the *ppsA* gene (Rv2931) E419K encoding for a polyketide synthase involved in the synthesis of phenolphthiocerol, also a part of PDIM molecules. The presence of these mutations in three different genes involved in PDIM biosynthesis indicates that PDIM levels must have a protective effect, when *alr* activity is affected. Likely, these modifications are making *M. tuberculosis* further impermeable to DCS. Consistent with these findings, it has been found that mutations in the *ppsA* gene offer a fitness benefit in XDR strains^40^. In addition, different mutations in the *ppsA*, *ppsB*, and *ppsC* genes have been implicated in pyrazinoic acid resistance in *M. bovis* BCG^41^. Although the mutations found by Gopal et al., and the ones described herein are not the same, none of our 11 DCS^R^ mutants showed any changes in the MIC_90_ to pyrazinamide or pyrazinoic acid (Fig. 1c; Supplementary Fig. 3). Interestingly, our fitness kinetics experiments reveal that there was no significant fitness cost associated with mutations causing DCS resistance in vitro.

### Some DCS^R^ mutants are less fit in human macrophages

We then examined the uptake by and survival of the DCS^R^ strains in human monocyte-derived macrophages. All DCS^R^ strains tested were taken up similarly by macrophages (Fig. 2b). DCS^R^4, bearing solely the D322N Alr mutation, was attenuated during infection of naive and of interferon-gamma-activated human macrophages, compared with the parent strain (Fig. 2b). The presence of the *mas* V1442G or the *ppsA* E419K SNPs led to compensatory benefits as strains DCS^R^1, DCS^R^2, DCS^R^3, DCS^R^7, DCS^R^8, DCS^R^9, DCS^R^10, and DCS^R^11 managed to infect, survive, and proliferate inside macrophages at similar levels compared with the parent strain (Fig. 2b). In contrast, DCS^R^5, bearing the deletion upstream of the *alr* gene and a SNP on *rv2962c* gene encoding a rhamnosyltransferase involved in PDIM biosynthesis^42^, was significantly attenuated for growth in human macrophages, compared with the parent strain (Fig. 2b). Similarly, DCS^R^6 was also attenuated in human macrophages, indicating an associated fitness cost with Rv2962c T234I and Alr D322N mutations. These data suggest that some of the SNPs found in DCS^R^ strains affect the ability of *M. tuberculosis* to grow inside human macrophages.

### Some DCS^R^ mutants are attenuated during mouse infection

We next chose one DCS^R^ strain representative of each group for analysis of fitness/attenuation in a mouse model of infection. In agreement with the results obtained in human macrophages, DCS^R^4 was significantly attenuated in the mouse model, compared with the parent strain, with a decrease in lung CFUs of more than two log_10_ during the chronic phase of infection (Fig. 2c). This failure to establish a high-burden infection in mice indicates that the Alr D322N mutation impairs *M. tuberculosis* cellular viability. The presence of the *mas* mutation (DCS^R^1) led to compensatory effects during the mouse infection as we observed before in macrophages. Unexpectedly, the presence of the *ppsA* mutation did not compensate in vivo, as seen with the mutant DCS^R^9 (Fig. 2c). No fitness compensation was observed with DCS^R^ strains having a mutation at the putative rhamnosyltransferase (Uniprot ID P9WN09), Rv2962c, including the *alr* repression mutant that was also found to be attenuated in mice (Fig. 2c). These results indicate that most of the DCS^R^ strains incur significant fitness cost in vivo that can be missed if only competition assays in vitro are used without the presence of stresses found in the host (Fig. 2d). In this case, clear compensatory mechanisms involving *mas* mutations but not the ones in *pssA* and *rv2962c* can reverse the observed fitness costs associated with DCS resistance in vivo.

### Overexpression of Alr causes DCS resistance

**Fig. 3:**
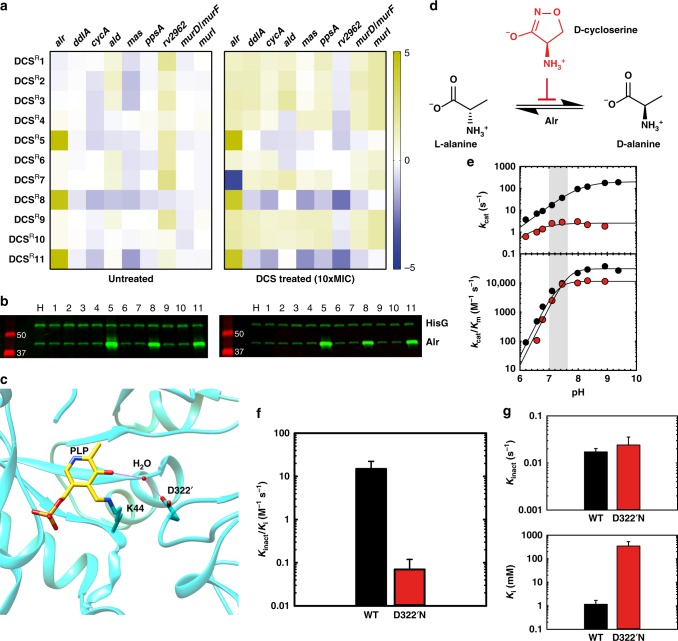
Cellular and molecular determinants of DCS resistance in *M. tuberculosis*. **a** qPCR results for differential gene expression (log_2_ transformed) of selected genes in the DCS^R^ mutants grown in the absence or presence of DCS (10 × MIC for 4 h). The results are representative of three biological replicates, *n* = 3. **b** Western blot analysis of Alr expression in the DCS^R^ mutants and parent strain grown in the presence or absence of DCS (10 × MIC for 4 h). HisG levels were used as loading control. The data are representative of two independent experiments. **c** Illustration of the active site of Alr from *M. tuberculosis*, 1XFC [https://www.rcsb.org/structure/1XFC] highlighting the location of residue D322 and its interactions with the pyridoxal 5’-phosphate cofactor through a water molecule-hydrogen bond network. **d** Reaction catalyzed by Alr and its inhibition by DCS. **e** pH-rate profile of the reaction catalyzed by wild-type and D322’N Alr. **f**, **g** Inactivation kinetics of wild-type (WT) and D322’N Alr by DCS at pH 7.5. Source data are provided as a Source Data file

In order to understand the molecular and cellular mechanisms involved in DCS resistance in TB, we first investigated the gene expression pattern of a selected panel of genes including *alr* and *ddlA*, encoding both targets of DCS. The DCS^R^5, DCS^R^8, and DCS^R^11 mutants that contained either mutations in the *alr* promoter or a deletion upstream of *alr* overexpressed *alr* gene by more than 30-fold when compared with the parent strain (Fig. 3a). This result indicates that the large deletion upstream *alr* found in DCS^R^5 likely removes a transcription factor binding site for an as yet unknown transcriptional repressor, but alternative mechanisms that lead to enhanced gene expression could also be involved. In contrast, all other DCS^R^ strains displayed similar levels of *alr* expression (Fig. 3a). There was no change in the expression of the *ddlA* gene in any strains indicating again that *ddlA* is not linked with DCS resistance in *M. tuberculosis*. Also, no changes in the RNA levels of genes related to downstream biochemical reactions in peptidoglycan biosynthesis such as *murD*, *murF*, and *murI* were observed in any of the DCS^R^ strains (Fig. 3a). In addition, during an acute DCS treatment (high dose, short time), transcriptional changes of these selected genes were partially altered in the majority of the mutants. There is upregulation of DCS targets, *alr* and *ddlA*, as well as *cycA* and the downstream peptidoglycan biosynthesis-related genes *murD*, *murF*, and *murI* (Fig. 3a). These subtler transcriptional changes likely indicate a concerted effort to maintain the structural integrity of peptidoglycan in *M. tuberculosis* under DCS stress (Fig. 3a). We further examined Alr protein levels, using a specific anti-Alr serum, and found that Alr is also elevated in the DCS^R^5, DCS^R^8, and DCS^R^11 strains (Fig. 3b), consistent with the increase in *alr* RNA levels observed in Fig. 3a. Alr overexpression in DCS^R^5, DCS^R^8, and DCS^R^11 was also observed during acute exposure to DCS. Increased levels of Alr may serve the cell in two ways during DCS exposure: (i) providing extra D-Ala which will protect Ddl from DCS inhibition and (ii) removing free DCS from the cell, functioning as a “sponge”, a mechanism already shown in aminoglycoside resistance^43^. Taken together, these results indicate that overexpression of Alr alone is sufficient to cause DCS resistance in *M. tuberculosis*.

### DCS inhibition is significantly reduced on the Alr D322’N

We next investigated the effect of the D322N substitution on Alr activity, as this mutation was present in eight out of the 11 mutants isolated (~70% of the mutants). Analysis of the structure of *M. tuberculosis* Alr indicates that D322 from the adjacent monomer makes a water-mediated hydrogen bond with the pyridoxal 5′-phosphate cofactor (Fig. 3c). As such we will refer to it hereafter as D322’. Such an interaction suggests roles for this residue in either ligand binding, catalysis, and/or inhibition. Recombinant Alr (wild-type) and Alr D322’N were expressed, purified and enzymatic activity as well as inhibition by DCS were evaluated (Methods and Fig. 3d–g). We first analyzed the pH profile of Ala racemization carried out by wild-type and the D322’N mutant. Under *k*_cat_ conditions, the D322’N mutant has significantly lower catalytic activity compared with wild-type Alr across all pH values tested (Fig.3e). At neutral pH, Alr D322’N is ~20-fold slower than wild-type enzyme. Under *k*_cat_/*K*_m_ conditions, no differences were observed for wild-type and D322’N Alr enzymes in the pH range tested. As DCS forms a covalent inhibitor with alanine racemases pyridoxal 5′-phosphate cofactor and IC_50_ measurements are misleading for irreversible inhibitors, we evaluated *k*_inact_ and *K*_i_ parameters for wild-type and D322’N enzymes with DCS. The D322’N point mutation in Alr leads to a 240-fold decrease in the *k*_inact_/*K*_i_ for DCS (Fig. 3g). This result indicates that despite the moderate impact under *k*_cat_ conditions, Alr D322’N is highly resistant to DCS inhibition. Separation of *k*_inact_ (rate of inactivation) and *K*_i_ (binding constant) parameters revealed that the D322’N mutation leads to a loss of affinity for DCS (Fig. 3f), with only a very modest increase in the inactivation rate (Fig. 3f). Together, these results indicate that the most common mechanism to generate DCS resistance is by the modification of Alr, decreasing its affinity for DCS. Decreased affinity for DCS would lead to more alanine racemisation, which in turn would protect Ddl from inhibition, as DCS is a competitive inhibitor against D-alanine.

## Discussion

Prediction of resistance to novel antibiotics and pre-engineering/selection of compounds with ultra-low rates of emergence of resistance is essential for modern antibiotic development^44^. Two more fundamental characteristics when evaluating drug resistance are the inherent rate to which mutations conferring resistance can arise in a particular pathogen, and the potential ways in which fitness cost can be mitigated by alternative or compensatory mechanisms. The rate of spontaneous mutations conferring resistance to DCS (mutation rate) is ultra-low in *M. tuberculosis* (ca. 10^−11^) (ref. ^33^ and this work). Mutation rate is important, but identifying and understanding potential routes for compensating fitness costs associated with resistance are equally important to evaluate and predict resistance appearance and dynamics in populations^44^. Estimation of fitness costs not only in bacterial growth medium but also in cellular and whole-organism models is essential to uncover the true likelihood of a genotype to be propagated or eliminated from a population. We found that generation of mutants in vitro is an ideal way to access high variability of genotypes, which can then be studied in various models. This contrasts with the use of clinical strains, which have already been selected to allow growth in the host and therefore will only have low to negligible fitness costs^45^. In addition, we found poor correlation between fitness measured in growth medium with fitness measured in macrophages and mice. In vitro estimations of bacterial fitness, which are more commonly carried out in a laboratory setting, seem to significantly underestimate phenotypes that are clearly observed in cellular or whole-organism models. Moreover, we validated that the macrophage model of infection might be a more reliable surrogate to evaluate fitness costs in vitro compared with conventional competition assays in microbiological media (Fig. 2d).

This study is in agreement with studies highlighting the implication of *alr* in DCS resistance in *M. tuberculosis*^22,23^. We showed that there are two *alr*-related mechanisms underlying DCS resistance: overexpression of Alr or reduction of the inhibition of Alr through a previously unknown single-point mutation. We showed that a promoter mutation upregulates *alr* gene transcription and increases Alr protein level up to 30-fold (Fig. 3a, b). Consistent with this mechanism, mutations in the *alr* promoter region have been found in clinical strains (position 3,841,356; G > A)^22,46^ in close proximity with our promoter mutation found in DCS^R^8 and DCS^R^11 strains (3,841,405; C > A) indicating a possible area for mutagenesis upstream of *alr* gene. In addition to this mutation, we also observed a deletion further upstream of the *alr* starting codon, possibly in an area where a yet-to-be-identified transcriptional repressor might bind. This deletion resulted in increased transcription of the *alr* gene with concomitant Alr overexpression, as observed in the promoter mutation strains. This mutation has not yet been found in the clinic. In agreement with our results, Alr overexpression was shown to be sufficient to cause DCS resistance in *M. tuberculosis*, where overexpression of Alr caused a sevenfold increase in the MIC of DCS^47^. The most common DCS resistance mechanism involves Alr modification (D322’N). This mutation reduces the affinity of Alr by DCS by over 240-fold, with some associated disruption of its catalytic activity. One XDR-TB strain contained a mutation on the preceding residue, M221T, implicating the importance of these two residues in the inhibition of Alr by DCS.

Furthermore, the fact that all DCS-resistant mechanisms presented here, also identified in clinical strains, involve mutations in the *alr* locus and not in the *ddlA* locus highlights the importance of Alr in generating DCS resistance. As both Alr and Ddl are part of sequential enzymatic reactions and mutations in the first gene, *alr*, will lead to a less inhibited Alr enzyme and thus increased levels of D-alanine will then out-compete DCS for the binding to the Ddl. This result is in agreement with our previous findings implicating Ddl and dipeptide synthesis as the lethal target of DCS in *M. tuberculosis*. Mutation to *ddlA* gene could confer DCS resistance, but it will not protect Alr from being inhibited by DCS. Therefore, we believe that synthesis of the dipeptide is the key for antibiotic mediated killing, while mutations on the *alr* gene protect both targets.

In summary, DCS resistance is an ultra-rare event in *M. tuberculosis* and secondary compensatory mutations, such as the ones observed in the PDIM biosynthetic pathway, might alleviate potential fitness costs. Therefore, the lack of widespread clinical resistance to DCS is due to the low mutational rate of *M. tuberculosis*. DCS is known to inhibit two enzymes from the same pathway, and therefore it seems reasonable to propose that finding other molecules that truly inhibit more than one essential enzyme is the most rational route to resistance-evading antibiotics.

Lastly, the results of this work, in combination with clinical WGS studies and phenotypic data can help improve the diagnosis of DCS resistance through generation of molecular tools for mapping DCS-conferring mutations. Importantly, our findings can be utilized to improve the design of second-generation DCS analogues that will maintain low mutation frequency and avoid resistance while minimizing undesirable cytotoxic effects.

## Methods

### Materials

Unless otherwise stated, all the chemicals and reagents used in this study were purchased from Sigma-Aldrich.

### Bacterial strains and conditions

*Mycobacterium tuberculosis* laboratory strain H37Rv was routinely cultured in Middlebrook 7H9 broth (Difco) supplemented with either 10% (v/v) of ADC enrichment (Difco), 0.05% (v/v) tyloxapol, and 0.02% (v/v) glycerol or with 10% of (v/v) ADGNT solution (0.5% bovine serum albumin, 2% dextrose 2% glycerol, 0.85% NaCl, and 0.4% tyloxapol) (c7H9). Liquid cultures were grown at 37 °C either in 50 mL centrifugation tubes with rotation of 40 rpm or in roller bottles with a rotation of 2 rpm. For solid growth experiments, *M. tuberculosis* H37Rv was grown in Middlebrook 7H10 (Difco) or Middlebrook 7H11 (Difco) agar medium supplemented with 10% (v/v) of OADC enrichment (Difco) and 0.5% (v/v) glycerol (c7H10 or c7H11).

### Calculation of DCS mutation rates

Mutation rates were calculated using the fluctuation assay and the Poison distribution methodology with few modifications^48^. Briefly, *M. tuberculosis*, starter cultures were inoculated from freezer stocks until the culture reached an OD_600_ of 1 and ~300,000 cells were used to inoculate 120 mL of c7H9, giving a total cell count of 10,000 cells per 4 mL culture. This volume was immediately divided to start 30 cultures of 4 mL each in 15 mL centrifugation tubes. Cultures were grown at 37 °C with rotation of 40 rpm until saturation (around 4 weeks). Following this, 26 cultures were spun at 3220 × g for 10 min at 4 °C. Cultures were then resuspended in 250–500 μL of 7H9 media and spotted onto multiwell plates containing c7H10 agar medium supplemented with the drug under investigation (DCS at 64 and 100 µg mL^−1^, RIF at 2 µg mL^−1^, and INH at 1 µg mL^−1^). Once spread plates were allowed to dry and were subsequently incubated at 37 °C for 1 month. Cell counts were determined by serial dilution of four remaining random cultures. Following that, the mutation rates can be calculated using the following formulas:

For the proportion (*P*_*0*_) of the cultures with no mutants

1 $$P_0 = \frac{{{\mathrm{no.}}\;{\mathrm{of}}\;{\mathrm{cultures}}\;{\mathrm{with}}\;{\mathrm{no}}\;{\mathrm{mutants/growth}}}}{{{\mathrm{total}}\;{\mathrm{no.}}\;{\mathrm{of}}\;{\mathrm{cultures}}\,\left( {\mathrm{i.e.}}, 26 \right)}}$$

For the number of mutation per culture (*m*)

2 $$m = - \ln \left( {P_0} \right)$$

Finally for the mutation rate (*μ*)

3 $$\mu = \frac{m}{{{\mathrm{total}}\;{\mathrm{CFUs}}}}$$

### Isolation of spontaneous D-cycloserine-resistant mutants

A fresh culture of *M. tuberculosis* H37Rv was set up in 5 ml c7H9 and incubated shaking at 37 °C until mid-exponential phase. The optical density was recorded and the culture diluted to a final OD_600_ of 0.000005 (~500 cells mL^−1^) in a total volume of 200 mL c7H9. Aliquots were taken and spread on 7H11 plates for CFU analysis of initial culture concentration. In total, 4 mL aliquots were then transferred into 60 individual 15 mL sterile polystyrene culture tubes, and incubated with rotation at 37 °C until saturation (~1 month). Aliquots were taken from select tubes and plated for CFU analysis of final culture concentrations. Tube contents were then inoculated, in full, onto individual 47 mm 0.22 -µm nitrocellulose membrane filters by vacuum, and the resulting bacteria-laden filters were transferred to individual c7H10 plates containing DCS at 100 µg mL^−1^ (5 × MIC). Plates were incubated at 37 °C, with filters transferred to fresh plates every 8–9 days for a period of 5 to 7 weeks. Individual colonies were then picked and inoculated into fresh c7H9, and freezer stocks generated. In addition, potential mutants were also spreaded on c7H10 plates containing 100 µg mL^−1^ of DCS to verify heritable acquisition of DCS resistance.

### Minimum inhibition concentration assays

Cultures were inoculated to a final OD_600_ of 0.001 into 100 µL of 7H9 in 96-well microtiter plates, with twofold serial dilutions of test compound going down each row (final column left without any drug). Top concentrations were chosen on a per-drug basis. Plates were incubated at 37 °C for 6 days, and then a solution containing 0.02% of resazurin dye was added on all the wells and incubated for another day. Minimum inhibition concentrations (MIC_90_) was calculated using visual determination of the lowest concentration of drug at which there was no change in the resazurin color.

Determination of MICs were also performed using the automated BD BACTEC™ MGIT™ 960 Instrument. Positive MGIT tubes containing the DCS^R^ and parent wild-type strains were used to inoculate new tubes containing DCS at final concentrations of 0, 2, 4, 8, 16, 32, 64, and 128, µg mL^−1^, in duplicates. In the MGIT, the lowest concentration of DCS that prevented the generation of florescence from the DCS-containing tube within 2 days of the no drug control tube was defined as the MIC.

### Whole-genome sequencing

In all, 2 mL of stationary phase cultures of desired strains were harvested by centrifugation, resuspended in 0.2 mL TE buffer, and heat-killed at 80 °C for 1 h. Genomic DNA was extracted using the Zymo gDNA extraction and genomic DNA clean and concentrator kits, following the manufacturer’s protocols. Quality and quantity of genomic DNA were confirmed by agarose gel electrophoresis, and DNA sequencing performed on an Illumina HiSeq 2000 instrument at the Cricks Genomic Science Technology Platform.

### Mapping genome data and SNP calling

Sequencing reads for the WT and the 11 DCS^R^ mutant strains were mapped against the *M. tuberculosis* H37Rv reference genome (AL123456) as single-end data using the Burrows-Wheeler algorithm described in BWA^49^ (Supplementary Data 1). Mapping outputs were used to generate SNP calls using SAMtools^49^. Filtering of SNP calls was performed by keeping those SNPs with minimum mapping quality of 10 and maximum read depth of 400. These high confidence calls were used to generate a nonredundant list of variable positions called in at least one strain and used to recover the base call in all other strains. Finally, SNPs positions that involved heterozygous calls or felt in a repetitive or mobile element were removed. Functional annotation of each of the polymorphic positions identified was performed using ANNOVAR^50^. The SNPs identified are listed in Supplementary Data 2. All genome data is available in ArrayExpress under accession number E-MTAB-5935.

### Sanger sequencing

DNA fragments including the identified SNPs were amplified via PCR with specific primers outlined in Supplementary Data 4, PCR amplicons were then purified using a Qiagen PCR clean up kit and then sent for Sanger sequencing at GATC Ltd (Germany). Sequencing files were visualized and compared to wild-type using the Unipro UGENE software.

### Competition assays

*M. tuberculosis* H37Rv parent strain and all DCS-resistant mutants were grown at 37 °C in c7H9 media until late logarithmic phase separately in roller bottles. The cultures were then diluted and a new set of cultures initiated by co-culturing together the parent strain and each of the DCS-resistant mutant at an equal ratio (2 × 10^5^ CFUs) and incubated over the course of the competition assay for 56 days. An aliquot of the mixed culture was taken at the following timepoints, 0, 7, 14, 28, and 56 days, serially diluted and plated in triplicate in c7H10 plates with and without 30 µg mL^−1^ of DCS for CFU determination. CFUs were counted following 1 month of incubation at 37 °C. Three independent experiments were performed with duplicate cultures in each of them. The relative fitness of the DCS susceptible over the DCS-resistant strains was calculated using the formula:

4 $$W = {\mathrm{ln}}(\frac{{R_e}}{{R_b}}) \div {\mathrm{ln}}(\frac{{S_e}}{{S_b}})$$

Where R_e_ and S_e_ are the number of resistant and susceptible bacilli at the endpoint (7, 14, 28, and 56 days), respectively, and R_b_ and S_b_ are the number of resistant and susceptible bacilli at the baseline, time 0.

### Infection of human monocyte-derived macrophages

White blood cells were isolated from leukocyte cones (NC24) supplied by the NHS Blood and Transplant service, UK and complied with the UK Human Tissue Act regulations. GM-CSF-derived macrophages were plated in 24-well tissue culture plates at a final concentration of 2 × 10^5^ cells per well 1 day prior to infection. *M. tuberculosis* cultures were grown to the mid-log phase (OD_600_ ~0.8). Prior to infection, *M. tuberculosis* cultures were harvested by centrifugation at 3000 rpm for 5 min and washed once with PBS and once with cell culture media (RPMI with 10% FCS). An equal volume of sterile glass beads (2.5–3.5 mm) that matched the pellet size was added and vigorously shaken for 1 min to make single-cell suspension. The bacteria were then resuspended in cell culture media and spun at 1200 rpm for 5 min. Following centrifugation, the supernatant containing single-bacterial cells was transferred into a new tube, OD_600_ was measured and the culture was diluted accordingly to 0.1 OD to make the infection solution (~8 × 10^8^ cells mL^−1^). Macrophages were then washed and suspended with 0.25 mL per well of the infection solution (MOI of 1) for 2 h. Following infection, macrophages were then washed three times with PBS and suspended with cell culture media for the course of infection.

### CFU determination

For counting bacterial viability, macrophages were washed once with PBS to remove extracellular bacteria and lysed with 0.5 mL of water–Tween-80 (0.05%) per well for 1 h at room temperature. The lysed solution from the triplicate wells was then used for serial tenfold dilutions in PBS–Tween-80 (0.05%). In all, 20 µL from each dilution was then plated in triplicate onto c7H11 agar plates. Agar plates were incubated for 3–4 weeks at 37 °C. CFU counts were calculated and plotted as the mean CFU per mL.

### Mouse infections

C57BL/6J (WT) mice were bred and maintained under specific pathogen-free conditions at The Francis Crick Institute, Mill Hill Lab or purchased from Charles River. All studies were ethically reviewed and approved by the respective ethical review committees at the Francis Crick Institute and NIBSC. Procedures involving mice were performed in strict accordance with the United Kingdom Animals (Scientific Procedures) Act 1986 and the respective Institutes policies on the Care, Welfare and Treatment of Animals. Procedures were done under UK Home Office animal licenses 70/8045 and P7611793C. Animals were monitored by trained animal technicians at least once a day, and this frequency could increase if any adverse reactions were observed. Infections were performed in the containment level 3 animal facilities either at the Mill Hill Lab or NIBSC. For mouse infections, *M. tuberculosis* H37Rv and DCS-resistant strains were cultured in c7H9 broth, to an OD_600_ of 0.6. From this, an infection sample was prepared to enable delivery of about 100 CFUs/mouse lung using a nebulizer system (Walkers, UK) linked to a Middlebrook airborne infection device (Glas-col, Terre Haute, USA). Infection was monitored by assessing homogenized lungs from infected mice at defined time intervals. Bacterial counts were determined by plating serial dilutions of homogenates on duplicate c7H11. CFUs were counted 2–3 weeks after incubation at 37 °C. The data at each timepoint are the means of five mice per group ± standard error (SEM).

### RNA extraction and quantitative qPCR

Early logarithmic phase cultures of *M. tuberculosis* and DCS-resistant mutants were grown in triplicates and then either treated with 10 × MIC of DCS or carrier control for 4 h. Following treatment, cultures were spun down at 3000 × g for 5 min at 4 °C, supernatant discarded and suspended in 1 mL of Trizol reagent and transferred into a 2 -mL tube containing lysing matrix B beads (MP Biomedicals). Samples were lysed using a ribolyser at 6.5 speed for 45 s twice. Following ribolysis, samples were spun down, and the RNA was purified using the Direct-zol^TM^−96 RNA kit (Zymo Research) following the manufacturer’s instructions with the on column DNase treatment. Following RNA purification, the RNA was further DNase treated by a vigorous treatment using the Ambion DNA Free kit according to the manufacturer’s instructions. RNA quantity and quality was estimated using NanoDrop and 1.5 µg of RNA was used to make cDNA according the High-Capacity RNA-to-DNA kit (Applied Biosystems). qPCRs were done using TaqMan probe assays to quantify the levels of a selection of genes (Supplementary Data 4) on a QuantStudio 5 Real-Time PCR System. PCR efficiencies and limit of detection were calculated for each TaqMAn assay using genomic DNA from *M. tuberculosis*. Differential gene expression was calculated using the cloud-based AB software.

### Western blotting

Early logarithmic phase cultures of *M. tuberculosis* and DCS-resistant mutants were grown in and either treated with 10 × MIC of DCS or carrier control for 4 h. Following treatment, the cultures were spun down at 3000 × g for 5 min at 4 °C, supernatant was discarded, and the pellet was washed twice with PBS solution and suspended into 1 mL of protein lysis buffer (20 mM TAE pH 7.8, 8 M urea, 10% glycerol and 5 mM TCEP (Tris[2-carboxyethyl]phosphine hydrochloride)) and transferred into a 2- mL tube containing lysing matrix B beads (MP Biomedicals). Bacterial suspensions were lysed using a ribolyser at 6.5 speed, 45 s, three times with intermittent cooling on ice, followed by centrifugation at top speed for 10 min in a refrigerated microcentrifuge. The supernatants containing soluble protein fractions were then passed through a 0.22 µm pore-size spin filter, and stored at −80 °C until further use. Thirty micrograms of protein of each sample were run per lane of a SDS-PAGE gel alongside Odyssey® One-Color Protein Molecular Weight Markers (Li-Cor Bioscience) and blotted to polyvinylidine fluoride membranes using iBlot 2 Dry Blotting System (ThermoFisher Scientific). Membranes were blocked using the Odyssey Blocking buffer (Li-Cor Bioscience) for 2 h at room temperature and subsequently probed with primary rabbit-raised anti-MtAlr antibody (1:5000 dilution) overnight at 4 °C. Following washing, membranes were incubated for 1 h with IRDye 800CW Goat anti-Rabbit IgG (Li-Cor Bioscience) secondary antibody, and the immunoblots were developed using the Odyssey CLx Imaging System (Li-Cor Bioscience).

### Recombinant Alr purification

MtAlr D322N was generated using QuickChange mutagenesis (Agilent; cloning primers; Supplementary Data 4), and pET28:MtAlr as a template. Recombinant proteins (including *Escherichia coli* L-alanine dehydrogenase; EcLADH) were expressed in *E. coli* BL21(DE3) and purified by nickel-affinity chromatography^51^. Both enzymes were co-expressed with the mycobacterial and *E. coli* GroEL and GroES chaperone proteins, respectively. Purified histidine-tags were removed using thrombin (Restriction grade, GE Healthcare) and dialyzed three times to remove the cleaved tags in 20 mM TEA or 20 mM NaPO_4_, 150 mM NaCl, pH 7.8, concentrated, flash frozen, and stored at −80 °C until activity assays.

### MtAlr activity measurements

Alanine racemase activity was measured in the L-Ala to D-Ala direction^51^. Briefly, reactions containing 50 mM buffer (see below), 5 mM α-ketogluratate, 0.25 mM NADH, 18–28 U ml-1 LDH (used as a mixture of pyruvate kinase/lactate dehydrogenase for convenience; Sigma), 4 μM BsDAAT, and varying concentrations of L-Ala were combined with 10–100 nM recombinant enzyme and monitored at 340 nm. Buffers used for each pH were HEPES (7.62, 7.12), Tris (7.92), TAPS (8.18), MOPS (6.86), CHES (8.9), PIPES (6.57), and MES (6.3). All reactions were performed at 37 °C. *K*_*m*_ and *k*_*cat*_ values were calculated by fitting data to the Michaelis–Menten equation.

DCS inactivation reactions comprised 5 µM recombinant Alr, 20 mM sodium phosphate buffer (pH 7.5 or 6.85), 150 mM NaCl, and varying concentrations of DCS. Reactions were commenced upon addition of DCS. At distinct timepoints during the incubation (including a time 0, before DCS was added), aliquots were removed and added to activity reactions, comprising 50 mM CHES pH 8.9, 5 mM NAD^+^, 20 mM D-Ala, and 30 µL mL^−1^ *Ec*LADH. Activity reaction progression was monitored at 340 nm.

Activity at time *t* was re-calculated as a percentage of the initial activity of the enzyme (activity at *t*_*0*_). Plots of *ln*(% activity) vs. time were linear in all cases. The slope of each plot allowed calculation of the *k*_obs_ for each inhibitor concentration tested, per Eqs. (1) and (2):

5 $$V(t)/V(0) = a \times e^{\left( { - k_{obs} \times t} \right)}$$

6 $$ln\left( {V(t)/V(0)} \right) = k_{obs} \times t + a$$

Where *a* is a *Y*-axis normalization factor, *V(*_*t*_*)* is activity at time *t*, and *V(*_*0*_*)* is activity at time 0. Nonlinear regression analysis of replots of *k*_*obs*_ *vs*. inhibitor concentration allowed calculation of *k*_inact_ and *K*_*i*_, per Eq. (3):

7 $$k_{obs} = \left( {k_{inact} \times \left[ I \right]} \right)/\left( {K_i + \left[ I \right]} \right)$$

### Lipid extraction

Early logarithmic phase cultures of *M. tuberculosis* and DCS-resistant mutants were grown for lipid profiling. Apolar lipids were extracted following established procedures as previously described^52^. Briefly, 20 mL of *M. tuberculosis* H37Rv and DCS-resistant strains were cultured in c7H9 broth, to an OD_600_ of 2, harvested via centrifugation at 3000 × *g* for 5 min and transferred to 1 mL of PBS solution in a 2 mL screw cap tubes and heat inactivated at 90 °C for 2 h. Lipids were extracted from the remaining biomass in a mixture of methanol: chloroform (2:1) overnight, followed by a second extraction of methanol: chloroform (1:1) for 5 h at room temperature. Pooled lipid mixture was then dried at 50 °C under a nitrogen stream. Extracted lipids were dissolved in chloroform and subsequently analyzed using thin layer chromatography. The extracted lipids were then analyzed using two-dimensional TLC with system A solvents consisting of petroleum ether–ethyl acetate (98:2), three times in the first direction and petroleum ether–acetone (98:2), once in the second direction. TLC plates were visualized using a 5% phosphomolybdic acid solution in ethanol followed by gentle charring of the plates with a heat-gun.

### Statistical analysis

GraphPad Prism 7 was used for all statistical analysis. Three independent experiments per assay were performed and statistical analysis was carried out by unpaired *t* test of the biological replicates of each experiment.

### Reporting summary

Further information on research design is available in the Nature Research Reporting Summary linked to this article.

## Supplementary information

Supplementary Information

Description of Additional Supplementary Files

Supplementary Data 1

Supplementary Data 2

Supplementary Data 3

Supplementary Data 4

Reporting Summary

Source Data

## Data Availability

Whole-genome sequencing data of the strains used in this study are available in ArrayExpress (www.ebi.ac.uk/arrayexpress) under accession number E-MTAB-5935. The data underlying Figs. 1, 2, and 3 are provided in the Source Data file. All other data are available from the corresponding author upon reasonable requests.
