# Supplementary material for: Comparative fitness analysis of D-cycloserine resistant mutants reveals both fitness-neutral and high-fitness cost genotypes
Source: Nat Commun. 2019 Sep 13;10:4177. doi: 10.1038/s41467-019-12074-z (PMC6744398; doi:10.1038/s41467-019-12074-z)
Supplement: Supplementary file 1 — Supplementary Information [file 41467_2019_12074_MOESM1_ESM.docx]

Supplementary Information

Comparative fitness analysis of D-cycloserine resistant mutants reveals both fitness-neutral and high-fitness cost genotypes

Dimitrios Evangelopoulos^1^†, Gareth A. Prosser^1^†‡, Angela Rodgers^1^, Belinda M. Dagg^2^, Bhagwati Khatri^2^, Mei Mei Ho^2^, Maximiliano G. Gutierrez^3^, Teresa Cortes^4^, and Luiz Pedro S. de Carvalho^1^*.

^1^Mycobacterial Metabolism and Antibiotic Research Laboratory, The Francis Crick Institute, 1 Midland Road, London NW1 1AT, United Kingdom.

^2^Bacteriology Division, National Institute for Biological Standards and Control (MHRA-NIBSC), Blanche Lane, South Mimms, Potters Bar, Herts EN6 3QG, United Kingdom

^3^Host-Pathogen Interactions in Tuberculosis Laboratory, The Francis Crick Institute, 1 Midland Road, London NW1 1AT, United Kingdom.

^4^Department of Pathogen Molecular Biology, Faculty of Infectious and Tropical Diseases, London School of Hygiene and Tropical Medicine, London WC1E 7HT, United Kingdom.

†These authors contributed equally to this work.

*Correspondence to: Luiz P. S. Carvalho. Email: [luiz.carvalho@crick.ac.uk](mailto:luiz.carvalho@crick.ac.uk)

**This PDF file includes:**

Figs. S1 to S3


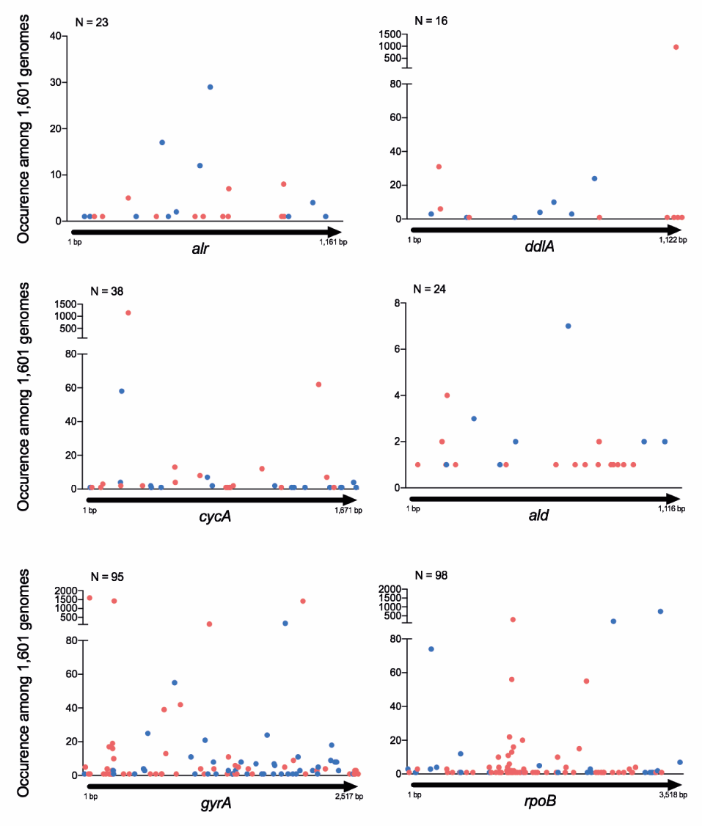


Fig. S 1 | Distribution and frequency of SNPs in DCS gene targets among a dataset comprising 1,601 *M. tuberculosis* clinical isolates. WGS from 1,601 M tuberculosis clinical isolates (*7*) were used to look at the type and frequency of SNPs described in the DCS target genes, *alr*, *ddlA*, *cycA* and *ald*. The genes *gyrA* and *rpoB* associated with drug resistance are also included as a mean of comparison. For each gene, synonymous changes are indicated in blue and non-synonymous changes are indicated in red.


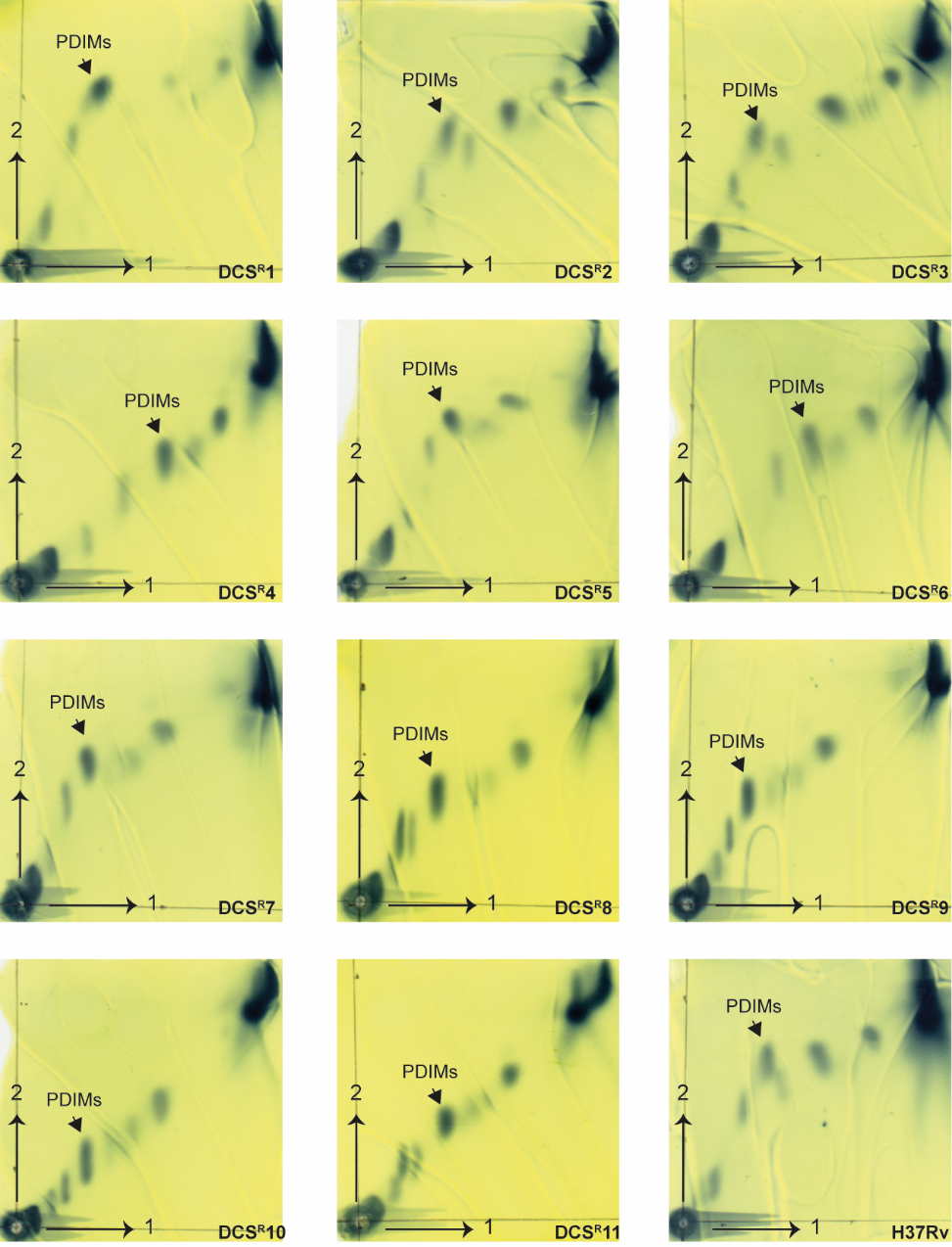


Fig. S 2 | Two-dimensional TLC analysis of lipid profiles of DCS resistant mutants. For the analysis of the apolar lipids including PDIMs, equal amounts of lipids extracted from *M. tuberculosis* H37Rv and DCS resistant mutants were loaded and developed with petroleum ether-ethyl acetate (98:3) in the first dimension, three times, and petroleum ether-acetone (98:2) in the second dimension once (TLC system A (*6*)). Lipids on the TLC plates were visualized using a 5% ethanolic solution of phosphomolybdic acid, following by gentle charring using a heat-gun. The PDIMs and the TLC dimensions are annotated on the plates.


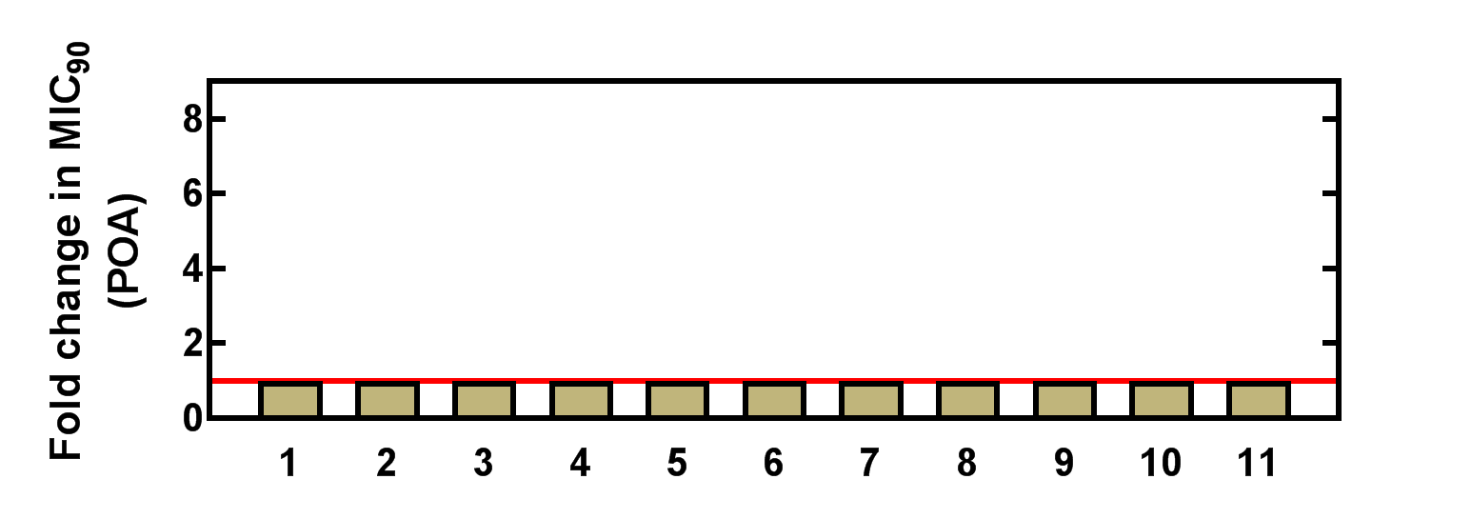


Fig. S 3 | Drug susceptibility pattern of pyrazinoic acid (POA) against DCS resistant mutants (MIC_90_ fold change DCS^R^/parent strain). Drug abbreviations used, POA; pyrazinoic acid. The MIC_90_ of the POA for the parent, DCS^S^ strain was 125 µg/mL.
