# Supplementary material for: Comparative fitness analysis of D-cycloserine resistant mutants reveals both fitness-neutral and high-fitness cost genotypes
Source: Nat Commun. 2019 Sep 13;10:4177. doi: 10.1038/s41467-019-12074-z (PMC6744398; doi:10.1038/s41467-019-12074-z)
Supplement: Supplementary file 2 — Description of Additional Supplementary Files [file 41467_2019_12074_MOESM2_ESM.docx]

**Description of Supplementary Files**

**File Name: Supplementary Data 1**

**Description:** List of single nucleotide positions and their occurrence identified on drug gene targets in Coll et al. 2014[DOI: 10.1038/ncomms5812].

**File Name: Supplementary Data 2**

**Description:** Mapping statistics for whole genome sequenced samples.

**File Name: Supplementary Data 3**

**Description:** List of variable single nucleotide positions identified among DCS resistant strains.

**File Name: Supplementary Data 4**

**Description:** List of primers and probes used in this study.
