# Supplementary material for: Comparative fitness analysis of D-cycloserine resistant mutants reveals both fitness-neutral and high-fitness cost genotypes
Source: Nat Commun. 2019 Sep 13;10:4177. doi: 10.1038/s41467-019-12074-z (PMC6744398; doi:10.1038/s41467-019-12074-z)
Supplement: Supplementary file 7 — Reporting Summary [file 41467_2019_12074_MOESM7_ESM.pdf]

nature research

Corresponding author(s): Luiz Pedro S. de Carvalho

Last updated by author(s): Jul 25, 2019

Reporting Summary

Nature Research wishes to improve the reproducibility of the work that we publish. This form provides structure for consistency and transparency in reporting. For further information on Nature Research policies, see Authors & Referees and the Editorial Policy Checklist.

Statistics

For all statistical analyses, confirm that the following items are present in the figure legend, table legend, main text, or Methods section.

n/a	Confirmed	
☐	☒	The exact sample size (*n*) for each experimental group/condition, given as a discrete number and unit of measurement
☐	☒	A statement on whether measurements were taken from distinct samples or whether the same sample was measured repeatedly
☐	☒	The statistical test(s) used AND whether they are one- or two-sided<br>*Only common tests should be described solely by name; describe more complex techniques in the Methods section.*
☒	☐	A description of all covariates tested
☒	☐	A description of any assumptions or corrections, such as tests of normality and adjustment for multiple comparisons
☐	☒	A full description of the statistical parameters including central tendency (e.g. means) or other basic estimates (e.g. regression coefficient) AND variation (e.g. standard deviation) or associated estimates of uncertainty (e.g. confidence intervals)
☒	☐	For null hypothesis testing, the test statistic (e.g. *F*, *t*, *r*) with confidence intervals, effect sizes, degrees of freedom and *P* value noted<br>*Give P values as exact values whenever suitable.*
☒	☐	For Bayesian analysis, information on the choice of priors and Markov chain Monte Carlo settings
☒	☐	For hierarchical and complex designs, identification of the appropriate level for tests and full reporting of outcomes
☒	☐	Estimates of effect sizes (e.g. Cohen's *d*, Pearson's *r*), indicating how they were calculated

Our web collection on statistics for biologists contains articles on many of the points above.

Software and code

Policy information about availability of computer code

Data collection	DNA sequencing was performed on an Illumina HiSeq 2000 instrument at the Crick's Genomic Science Technology Platform.
Data analysis	As described in Methods: Sequencing reads for the WT and the 11 DCSr mutant strains were mapped against the M. tuberculosis H37Rv reference genome (AL123456) as single end data using the Burrows-Wheeler algorithm described in BWA (Table S1). Mapping outputs were used to generate SNP calls using SAMtools. Filtering of SNP calls was performed by keeping those SNPs with minimum mapping quality of 10 and maximum read depth of 400. These high confidence calls were used to generate a non-redundant list of variable positions called in at least one strain and used to recover the base call in all other strains. Finally, SNPs positions that involved heterozygous calls or felt in a repetitive or mobile element were removed. Functional annotation of each of the polymorphic positions identified was performed using ANNOVAR.

For manuscripts utilizing custom algorithms or software that are central to the research but not yet described in published literature, software must be made available to editors/reviewers. We strongly encourage code deposition in a community repository (e.g. GitHub). See the Nature Research guidelines for submitting code & software for further information.

Data

Policy information about availability of data

All manuscripts must include a data availability statement. This statement should provide the following information, where applicable:

- Accession codes, unique identifiers, or web links for publicly available datasets
- A list of figures that have associated raw data
- A description of any restrictions on data availability

Whole genome sequencing data of the strains used in this study is available in ArrayExpress (www.ebi.ac.uk/arrayexpress) under accession number E-MTAB-5935. Data underlying Figs 1, 2, and 3 are provided as Source Data files. All other data are available from the corresponding author upon reasonable requests.

Field-specific reporting

Please select the one below that is the best fit for your research. If you are not sure, read the appropriate sections before making your selection.

☒ Life sciences ☐ Behavioural & social sciences ☐ Ecological, evolutionary & environmental sciences

For a reference copy of the document with all sections, see nature.com/documents/nr-reporting-summary-flat.pdf

Life sciences study design

All studies must disclose on these points even when the disclosure is negative.

Sample size	No sample size calculation was performed. For the mouse experiments, five mice per group was used in order to provide significant statistical power as has been demonstrated in previous published experiments.
Data exclusions	No data were excluded from analyses
Replication	We have indicated the number of independent experiments performed in the figure legends.
Randomization	Randomization of the samples was not deem to be appropriate for this study.
Blinding	Blinding was not deem to be appropriate for this study.

Reporting for specific materials, systems and methods

We require information from authors about some types of materials, experimental systems and methods used in many studies. Here, indicate whether each material, system or method listed is relevant to your study. If you are not sure if a list item applies to your research, read the appropriate section before selecting a response.

Materials & experimental systems

n/a	Involved in the study	
☐	☒	Antibodies
☒	☐	Eukaryotic cell lines
☒	☐	Palaeontology
☐	☒	Animals and other organisms
☒	☐	Human research participants
☒	☐	Clinical data

Methods

n/a	Involved in the study	
☒	☐	ChIP-seq
☒	☐	Flow cytometry
☒	☐	MRI-based neuroimaging

Antibodies

Antibodies used	The anti-Alr and anti-HisG polyclonal primary antibodies were custom-made using Covalab S.A.S. (Villeurbanne, France) services. Antibodies were used in 1:5000 dilution.
Validation	Antibodies were validated against purified protein and lysates from Mtb bacteria.

Animals and other organisms

Policy information about studies involving animals; ARRIVE guidelines recommended for reporting animal research

Laboratory animals	C57BL/6J (WT) mice were either bred and maintained under specific pathogen-free conditions at The Francis Crick Institute, Mill Hill Lab or purchased from Charles River.
Wild animals	*Provide details on animals observed in or captured in the field; report species, sex and age where possible. Describe how animals were caught and transported and what happened to captive animals after the study (if killed, explain why and describe method; if released, say where and when) OR state that the study did not involve wild animals.*
Field-collected samples	*For laboratory work with field-collected samples, describe all relevant parameters such as housing, maintenance, temperature, photoperiod and end-of-experiment protocol OR state that the study did not involve samples collected from the field.*
Ethics oversight	All studies were ethically reviewed and approved by the respective ethical review committees at the Francis Crick Institute and NIBSC. Procedures involving mice were performed in strict accordance with the United Kingdom Animals (Scientific Procedures) Act 1986 and the respective Institutes policies on the Care, Welfare and Treatment of Animals. Procedures were done under UK Home Office animal licenses 70/8045 and P7611793C.

Note that full information on the approval of the study protocol must also be provided in the manuscript.
